# A Comprehensive Review of Detection Methods for *Staphylococcus aureus* and Its Enterotoxins in Food: From Traditional to Emerging Technologies

**DOI:** 10.3390/toxins17070319

**Published:** 2025-06-23

**Authors:** Assia Mairi, Nasir Adam Ibrahim, Takfarinas Idres, Abdelaziz Touati

**Affiliations:** 1 Laboratoire d’Ecologie Microbienne, Faculté des Sciences de la Nature et de la Vie (FSNV), Université de Bejaia, Bejaia 06000, Algeria; assia.mairi@univ-bejaia.dz (A.M.); abdelaziz.touati@univ-bejaia.dz (A.T.); 2 Department of Biology, College of Science, Imam Mohammad Ibn Saud Islamic University (IMSIU), Riyadh 13318, Saudi Arabia; naabdalneim@imamu.edu.sa; 3 Laboratory for Livestock Animal Production and Health Research, Rabie Bouchama National Veterinary School of Algiers, Issad ABBAS Street, BP 161 Oued Smar, Algiers 16059, Algeria

**Keywords:** foodborne pathogens, *Staphylococcus aureus*, staphylococcal enterotoxins, food poisoning, detection methods, food contamination, epidemiological tracking, outbreak surveillance

## Abstract

*Staphylococcus aureus* is a leading cause of foodborne intoxication globally, driven by its heat-stable enterotoxins (SEs), which pose significant public health risks. This review critically evaluates modern and traditional methodologies for detecting *S. aureus* and its enterotoxins in food matrices, emphasizing their principles, applications, and limitations. The review includes a dedicated section on sample preparation and pretreatment methods for diverse food substrates, addressing a critical gap in practical applications. Immunological techniques, including ELISA and lateral flow assays, offer rapid on-site screening but face matrix interference and variable sensitivity challenges. Molecular methods, such as PCR and isothermal amplification, provide high specificity and speed for bacterial and toxin gene detection but cannot confirm functional toxin production. Sequencing-based approaches (e.g., WGS and MLST) deliver unparalleled genetic resolution for outbreak tracing but require advanced infrastructure. Emerging biosensor technologies leverage nanomaterials and biorecognition elements for ultra-sensitive real-time detection, although scalability and matrix effects remain hurdles. Mass spectrometry (MALDI-TOF MS) ensures rapid species identification but depends on pre-isolated colonies. Traditional microbiological methods, while foundational, lack the precision and speed of molecular alternatives. The review underscores the necessity of context-driven method selection, balancing speed, sensitivity, and resource availability. Innovations in multiplexing, automation, AI-based methods, and integration of complementary techniques are highlighted as pivotal for advancing food safety surveillance. Standardized validation protocols and improved reporting of performance metrics are urgently needed to enhance cross-method comparability and reliability in outbreak settings.

## 1. Introduction

*Staphylococcus aureus* is a leading cause of foodborne intoxication worldwide, primarily due to its ability to produce heat-stable enterotoxins (SEs) that persist even after food processing. These toxins, such as SEA-SEE and newer variants (SEG, SEH, and SEI), are responsible for staphylococcal food poisoning (SFP), characterized by rapid-onset nausea, vomiting, and diarrhea [1]. The incubation period for SFP typically ranges from 30 min to 8 h, with an average of approximately 3 h, depending on the amount of toxin ingested and individual susceptibility [2]. Contamination often occurs through improper handling of ready-to-eat foods (e.g., dairy, meats, and salads), where *S. aureus* proliferates and releases toxins. Notably, the hands of food handlers are a significant source of contamination. A study conducted in Thailand found that 78% of *S. aureus* isolates were obtained from the hands of food handlers, highlighting the critical role of manual contact in the transmission of this pathogen [3]. The global burden of SFP underscores the critical need for rapid and accurate detection methods to mitigate outbreaks, trace contamination sources, and enforce food safety regulations [4].

**Figure 1:**
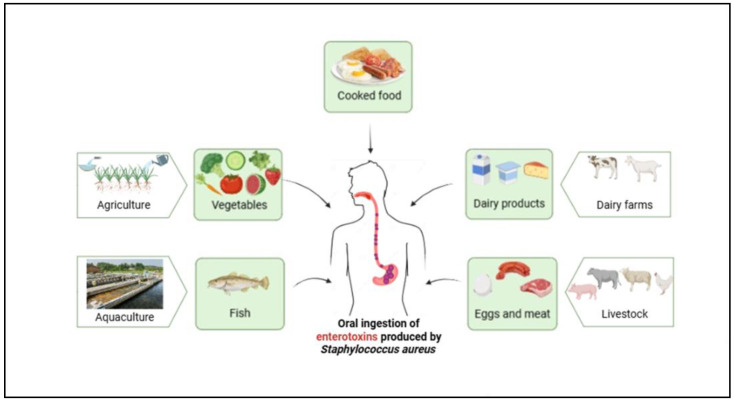
Food products contaminated by *Staphylococcus aureus*. This figure illustrates the pathways through which food products contaminated by enterotoxins produced by *S. aureus* can lead to oral ingestion. It highlights various sources of contamination, including cooked food, vegetables, fish, dairy products, eggs, meat, and livestock. The figure also emphasizes the role of agriculture, aquaculture, dairy farms, and livestock in potentially contaminating food products.

Food products contaminated by *S. aureus* and its enterotoxins, which can be ingested orally, are primarily sourced from various sectors, including agriculture (vegetables), aquaculture (fish), dairy farms (dairy products), livestock (eggs and meat), and cooked food. The ingestion of these enterotoxins can lead to foodborne illnesses (Figure 1) [5].

Early detection is pivotal in curbing the spread of toxigenic *S. aureus*, as even low toxin concentrations (≥20 ng) can induce illness [6]. Traditional culture-based methods, while foundational, are time-consuming and do not identify toxin-producing strains or methicillin-resistant *S. aureus* (MRSA), which remains a growing concern in food matrices. Molecular biology, immunology, and biosensing advances have revolutionized diagnostics, enabling high-throughput, specific, and sensitive identification of viable cells and enterotoxins. These methods are indispensable for public health responses, ensuring timely recalls, epidemiological investigations, and compliance with food safety standards [7].

**Figure 2:**
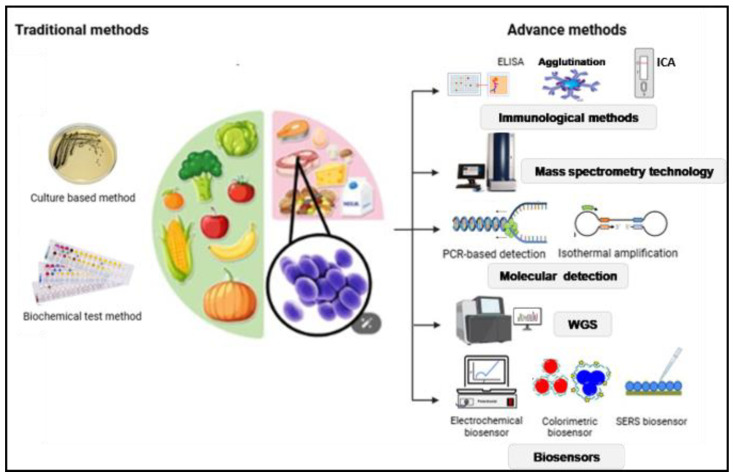
Traditional and modern approaches in *Staphylococcus aureus* detection in food. On the left, traditional methods include culture-based techniques, biochemical tests, and their application to food products. On the right, advanced methods involve immunological techniques (e.g., ELISA, agglutination, and ICA), molecular detection methods (e.g., PCR-based detection, isothermal amplification, and WGS), mass spectrometry technology, and biosensors (e.g., electrochemical, colorimetric, and SERS biosensors).

The detection landscape encompasses phenotypic (e.g., selective agar and antimicrobial susceptibility testing), immunological (e.g., ELISA and latex agglutination), molecular (e.g., PCR and whole-genome sequencing (WGS)), and emerging technologies (e.g., CRISPR-based assays and biosensors) (Figure 2). Each approach offers unique specificity, speed, or scalability advantages, tailored to diverse food matrices and outbreak scenarios. This review focuses on the research of enterotoxins as well as the broader microbiological diagnostics used to safeguard the food supply chain.

## 2. Sample Preparation and Pretreatment Methods for *Staphylococcus aureus* and Detection of Its Toxin in Food Matrices

Sample preparation for detecting *S. aureus* and its enterotoxins in dairy products, such as raw milk and cheeses, typically involves enrichment in Brain Heart Infusion (BHI) broth at 37 °C for 18–24 h, followed by centrifugation to separate the cells and supernatant. The supernatant, used for immunological detection methods like reversed passive latex agglutination (SET-RPLA), requires serial dilution (e.g., 100% down to 1.6%) to mitigate matrix interference [8,9]. For PCR-based detection of genetic targets, DNA extraction from the pelleted cells employs enzymatic pretreatment (e.g., lysostaphin and proteinase K) and boiling, or commercial kits like the Genomic Mini DNA Isolation Kit (NV, USA) or DNeasy tissue kit (Qiagen, Germany), prior to conventional or multiplex PCR [10,11]. Complex matrices like cheese often necessitate rigorous DNA extraction protocols to ensure PCR sensitivity [12]. For whole-genome sequencing (WGS) in dairy, selective enrichment precedes genomic DNA extraction using commercial kits from representative isolates [13].

For immunological enterotoxin detection in solid dairy matrices like cheese, intensive pretreatment is required: homogenization in distilled water, acidification to pH 3.5 ± 0.5 or pH 3.5–4.0 using HCl, centrifugation (e.g., 10,000× *g* for 15 min at 4 °C), neutralization to pH 7.3 ± 0.3 or pH 7.5 using NaOH, filtration through glass wool, and dialysis concentration against 30% polyethylene glycol (PEG 20,000) overnight at 4–5 ± 3 °C. The concentrated extract is adjusted to a defined weight (e.g., 5 g) using phosphate-buffered saline (PBS) before analysis with assays like RIDASCREEN^®^ SET Total (Germany) or VIDAS^®^ SET2 (Germany) [14,15]. Liquid matrices like milk or yogurt often allow simpler pretreatment, such as direct addition (100 µL) for ELISA or dilution prior to IgY-based Sandwich ELISA [16,17].

Ready-to-eat (RTE) foods, including bakery products, salads, and meats, frequently require homogenization (e.g., 25 g sample in 225 mL saline or buffered peptone water), pre-enrichment in BHI broth or buffered peptone water, and filtration (0.22 µm or 2.0 µm) of supernatants derived from centrifuged cultures. This prepares samples for immunological methods (SET-RPLA, VIDAS, and ELISA) or DNA extraction for PCR, qPCR, or LAMP [18,19,20]. For PCR-based detection in RTE foods, enrichment in BHI broth precedes DNA extraction via boiling and centrifugation, enabling subsequent PCR targeting genes like *nuc* [19]. Complex RTE foods or those with potential inhibitors may require DNA extraction using commercial kits (e.g., *Staphylococcus* sample preparation kit) after enrichment and filtration for reliable qPCR [18]. LAMP assays for meats or dairy utilize homogenization in phosphate-buffered dilution water, enrichment, and DNA extraction via Triton X-100 treatment and boiling for 10 min to generate crude templates [21].

Meat matrices (e.g., raw chicken, pork, and camel) employ homogenization, enrichment in broths like Trypticase Soy Broth (TSB) with 6.5% NaCl or double broth enrichment, and plating on selective media (e.g., Baird–Parker agar and Brilliance MRSA2) for phenotypic detection or colony isolation [22,23]. For PCR-SSCP genotyping, genomic DNA is extracted from isolates using standardized kits, followed by PCR amplification and denaturation of amplicons via heating and rapid cooling on ice to form single-stranded DNA conformations [24]. Cross-Priming Amplification (CPA) for starchy foods like frozen pastry involves artificial contamination of 25 g samples, DNA extraction, and pretreatment with Propidium Monoazide (PMA) to differentiate viable cells, enabling CPA amplification at 63 °C for 1 h targeting *femA* and *mecA* [25]. Powdered foods like milk powder require incubation in buffered peptone water, enrichment in BHI broth, and DNA extraction prior to CPA at 63 °C for 1 h [26]. MALDI-TOF MS identification uses protein extraction from colonies (e.g., using formic acid or 50% acetonitrile/1% trifluoroacetic acid) or direct spotting from selective agars like Baird–Parker, followed by matrix crystallization for spectral analysis [27,28,29].

## 3. Microbiological Methods

### 3.1. Objective and Methodology

The primary objectives of microbiological methods for detecting *S. aureus* in foodborne outbreaks are threefold: (1) to isolate and confirm the presence of *S. aureus* in diverse food matrices, (2) to evaluate AMR profiles critical for outbreak management, and (3) to assess virulence traits such as biofilm formation that influence pathogen persistence [30].

**Figure 3:**
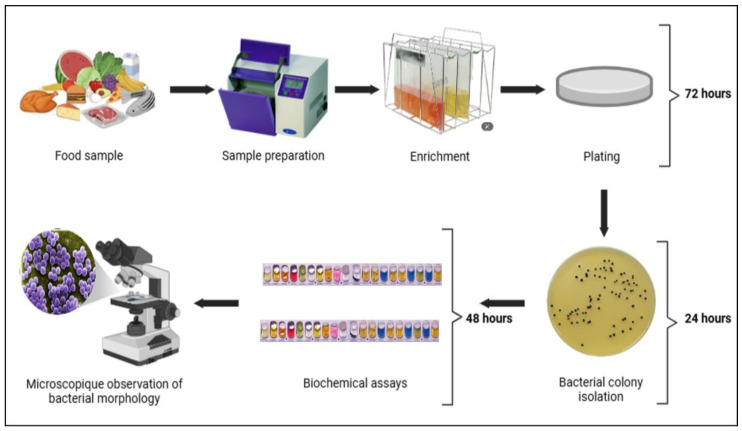
*Staphylococcus aureus* detection and isolation from food samples. This figure outlines the process of isolating *S. aureus* from food samples, starting with sample preparation and enrichment, followed by plating and microscopic observation, biochemical assays, and bacterial colony isolation.

The isolation of *S. aureus* is achieved through selective media tailored to its metabolic properties. Mannitol salt agar (MSA) is widely employed for food samples such as milk and beef, where *S. aureus* ferments mannitol, producing yellow colonies indicative of acid production. For instance, beef and milk samples are homogenized, plated on MSA, and incubated for 24 h, with subsequent biochemical or molecular confirmation [31]. Baird–Parker agar, supplemented with tellurite and egg yolk, offers enhanced selectivity for complex matrices like raw meats and dairy. The schematic in Figure 3 summarizes the detailed steps involved in this process. Colonies exhibiting proteolytic activity are further confirmed via PCR targeting the *nuc* gene, a thermonuclease specific to *S. aureus*, to distinguish it from coagulase-negative *Staphylococci* [32]. Chromogenic media, such as CHROMagar™ MRSA (France), streamline MRSA identification in dairy samples by producing pink to mauve colonies through enzymatic activity, reducing reliance on secondary tests [33].

AMR assessment aims to identify resistance patterns that guide treatment and containment strategies. As the Sensititre MRSA plate system (Lenexa, USA) exemplifies, broth microdilution inoculates bacterial isolates from foods like eggs or meat into multi-well plates containing graded antibiotic concentrations. The MIC is determined by observing growth inhibition, which is critical for detecting resistance to antibiotics such as oxacillin and clindamycin in MRSA strains [34,35]. E-test strips, applied to agar surfaces inoculated with isolates from milk or pork, generate gradient-dependent inhibition ellipses, enabling precise MIC determination for antibiotics like cefoxitin [36,37].

Disc diffusion methods, including the Kirby-Bauer technique, involve placing antibiotic-impregnated discs (e.g., penicillin and ampicillin) on Mueller–Hinton agar (Michigan, USA) inoculated with isolates from seafood, dairy, or vegetables. Inhibition zones are measured post-incubation and interpreted against CLSI guidelines to classify susceptibility. For example, seafood-derived *S. aureus* isolates showed high resistance to penicillin and ampicillin, with inhibition zones correlating to CLSI breakpoints [38,39].

Biofilm evaluation addresses the pathogen’s ability to persist in food processing environments. The microtiter plate (MTP) method cultures isolates from dairy or meat in 96-well plates, stains adherent biofilms with crystal violet, and quantifies biomass via optical density (OD570 or OD590). This semi-quantitative approach, applied to MRSA isolates from milk, demonstrated superior sensitivity in detecting weak biofilms compared to Congo Red Agar (CRA) (Rotherham, UK) [33,40].

Some studies have integrated multiple methods for robustness. For instance, Baird–Parker agar (Lansing, USA) isolation followed by PCR confirmation was used for *S. aureus* in camel and yak milk, ensuring specificity [32]. Similarly, combining disk diffusion with molecular methods (e.g., *mecA* gene detection) resolves discrepancies in phenotypic resistance interpretation, particularly for β-lactams [38,41].

### 3.2. Performance

The performance of microbiological methods for detecting *S. aureus* in foodborne outbreaks is evaluated through sensitivity, specificity, and comparative efficacy against alternative techniques, although reporting these metrics remains inconsistent. Sensitivity assessments revealed that disk diffusion achieved 100% sensitivity in identifying AMR among *S. aureus* isolates from bovine milk, where all samples exhibited resistance to at least one antibiotic [42]. Similarly, agar dilution methods demonstrated 100% sensitivity for oxacillin-resistant *S. aureus* (OS-MRSA) strains, with all isolates being resistant to ampicillin, penicillin, and cefoxitin [43]. Specificity improvements were noted when PCR confirmation of the *nuc* gene was applied to isolates from Baird–Parker agar, reducing the misidentification of coagulase-negative Staphylococci [32]. However, specificity varied significantly across studies, ranging from 100% for excluding MRSA in Koozeh cheese isolates [44] to as low as 1% in OS-MRSA evaluations, likely due to phenotypic heterogeneity [43].

Comparative analyses highlighted E-tests’ precision over traditional disk diffusion for antibiotics like cefoxitin and oxacillin, as gradient strips provided quantitative MIC values [36,37]. Broth microdilution (e.g., Sensititre systems) further identified multidrug resistance in 54% of bovine milk isolates, underscoring its utility for comprehensive resistance profiling [42]. Biofilm detection via the microtiter plate (MTP) method with crystal violet staining outperformed CRA in sensitivity, particularly for weak biofilm-forming MRSA strains from dairy samples. Chromogenic media such as CHROMagar™ MRSA (France) enabled rapid MRSA identification in dairy within 18–24 h, although explicit sensitivity and specificity data were omitted [33].

Method-specific strengths were evident in disk diffusion’s widespread adoption due to its simplicity and alignment with CLSI guidelines, despite having lower precision than molecular techniques. This method detected high resistance rates to penicillin (70–100%) in poultry and seafood isolates [38,39]. Selective media like MSA offered cost-effective isolation of *S. aureus* from milk and beef, although supplemental PCR was often required for confirmation [31]. Hybrid approaches, such as combining Baird–Parker agar with PCR, minimized false positives in camel and yak milk samples, demonstrating the value of integrating phenotypic and molecular methods [32].

### 3.3. Limitations/Challenges

The microbiological methods for detecting *S. aureus* in foodborne outbreaks face several limitations and challenges that impact their reliability, accessibility, and applicability. A significant issue is the inconsistent reporting of sensitivity and specificity metrics across studies. Many methods, such as Petrifilm™ Staph Express plates (Michigan, USA) and E-tests, lack quantitative performance data despite claims of rapid results, leaving their diagnostic reliability uncertain [36,45]. For instance, tetracycline resistance testing via disk diffusion reported no specificity data, limiting the interpretability of resistance patterns [46].

Antibiotic-specific variability further complicates resistance detection. Disk diffusion methods exhibit inconsistent sensitivity for β-lactams like penicillin and ampicillin, with resistance rates often under detected due to heteroresistance or methodological constraints [38,39]. Similarly, specificity fluctuated drastically in some studies, dropping to 1% for OS-MRSA strains, likely due to phenotypic heterogeneity or methodological limitations [43].

Matrix interference poses another challenge, particularly in complex food samples. High-fat dairy products and meat homogenates can inhibit bacterial growth on selective media or interfere with biofilm quantification in crystal violet assays, necessitating labor-intensive sample pretreatment [33,40]. Additionally, phenotypic methods like MSA lack specificity, often misidentifying coagulase-negative Staphylococci, which necessitates supplemental PCR confirmation [31,32].

Resource and training requirements limit the adoption of advanced techniques. Broth microdilution and PCR demand specialized equipment and expertise, restricting their use in resource-constrained settings [32,35]. Even widely used methods like disk diffusion suffer from subjectivity in interpreting inhibition zones, introducing variability between analysts [32,41]. Furthermore, time constraints affect outbreak responsiveness; while chromogenic media like CHROMagar™ MRSA reduce turnaround time, traditional culture-based methods remain slower, delaying critical interventions [33].

Finally, the lack of standardized comparative studies hinders the establishment of best practices. Few studies have directly contrasted methods such as E-test and broth microdilution beyond precision claims, leaving gaps in understanding their relative strengths [36,37]. These challenges underscore the need for standardized reporting, method validation across diverse matrices, and the integration of molecular techniques to enhance accuracy in outbreak settings [31,32].

## 4. Biochemical Methods

### 4.1. Objective and Methodology

Biochemical methods are designed to identify *S. aureus* and characterize its functional attributes through systematic analyses of metabolic, enzymatic, and physiological properties. The API Staph Test, for instance, employs a standardized panel of biochemical substrates to evaluate strain-specific metabolic reactions, such as carbohydrate fermentation and enzymatic hydrolysis. Bacterial isolates are inoculated into the test strips, and metabolic activity—evidenced by colorimetric changes or gas production—is interpreted against reference databases for species confirmation [37]. Automated systems like VITEK2 (France) and BD Phoenix (Phoenix, USA) enhance this approach by utilizing miniaturized biochemical panels to assess antibiotic susceptibility, substrate utilization, and hydrolysis patterns. These platforms integrate automated readers to generate biochemical profiles, which are algorithmically matched to reference libraries for rapid identification and resistance profiling. For example, the BD Phoenix System incorporates cefoxitin and oxacillin testing to detect methicillin resistance in *S. aureus* [45].

Enzymatic assays, such as the Coagulase Test, focus on detecting *S. aureus*’s ability to produce coagulase. Bacterial isolates are incubated in rabbit plasma, and fibrin clot formation is observed as a definitive marker for *S. aureus* identification [36]. Similarly, Baird–Parker agar combines selective and differential principles: samples are cultured on agar containing lithium chloride and tellurite to inhibit non-target bacteria, while *S. aureus* colonies are identified by their ability to hydrolyze egg yolk lipids (via lipase) and reduce tellurite, producing characteristic black colonies with clear zones. Post-incubation, colonies are visually inspected for morphology and proteolytic activity [47].

Toxin stability assessments evaluate the resilience of *S. aureus* enterotoxins (e.g., SEQ) under conditions mimicking food processing or digestion. Heat treatment involves exposing purified toxins to 100 °C and analyzing structural integrity via SDS-PAGE. In contrast, pepsin and trypsin treatments assess enzymatic resistance by incubating toxins with digestive enzymes and monitoring degradation patterns. These methods confirm toxin persistence, which is critical for understanding their role in foodborne outbreaks [48]. Selective culturing techniques, such as Baird–Parker agar, further support preliminary identification by combining biochemical interactions with morphological observations, enabling the differentiation of *S. aureus* from other *Staphylococci* without molecular tools [47].

### 4.2. Performance

Biochemical methods for detecting *S. aureus* exhibit variable sensitivity and specificity, with performance often dependent on the technique and target analyte. The API Staph Test demonstrates 75% sensitivity in agreement with PCR for identifying *S. aureus* in food and animal samples, although specificity data are not reported. This method provides “reasonably reliable phenotypic identification” compared to molecular techniques [37]. Automated systems like the VITEK2 achieve 100% sensitivity for MRSA detection in clinical isolates, offering rapid results compared to manual biochemical methods, although specificity remains unreported [45]. The BD Phoenix System is noted for its high-throughput capabilities, which enable efficient processing of clinical and food isolates, although sensitivity and specificity metrics are unspecified [45].

Traditional biochemical assays, such as the Coagulase Test, are widely used to confirm the presence of *S. aureus* in milk and dairy products but lack specific data. Their reliability is acknowledged, although they are less specific than PCR-based methods [36,47]. Similarly, Baird–Parker agar facilitates preliminary identification through selective culturing but is criticized for lower specificity than molecular approaches as it may misidentify coagulase-positive *Staphylococci* [47].

Methods assessing enterotoxin stability, such as heat, pepsin, and trypsin treatments, highlight the resilience of *S. aureus* enterotoxins (e.g., SEQ) under harsh conditions. SEQ exhibits strong heat resistance (retaining its structure at 100 °C) and protease stability, outperforming proteins like BSA and SEA in degradation assays. These findings underscore the persistence of toxins in food matrices, although sensitivity and specificity metrics for toxin detection are not provided [48].

### 4.3. Limitations/Challenges

Biochemical methods for detecting *S. aureus* face several constraints, primarily from their reliance on phenotypic traits and manual interpretation. A key limitation is subjectivity in visual assessments, particularly in methods like the Coagulase Test and Baird–Parker agar. For example, clot formation in coagulase testing and colony morphology on Baird–Parker agar require expert judgment, introducing variability and potential misidentification, especially when distinguishing *S. aureus* from other coagulase-positive *Staphylococci* [36,47].

Sensitivity and specificity gaps further challenge these methods. While the API Staph Test shows 75% agreement with PCR for sensitivity, specificity data are not reported, raising concerns about false positives [37]. Similarly, automated systems like VITEK2 and BD Phoenix, despite high sensitivity (e.g., 100% for MRSA detection), lack specificity metrics, limiting their reliability in mixed microbial environments [45].

Food matrix interference complicates biochemical assays, as components like fats, proteins, or competing microbiota may inhibit reactions or obscure results. For instance, Coagulase Tests and Baird–Parkeragar may underperform in complex samples such as dairy products or processed meats due to matrix-induced interference [47]. Toxin stability assessments (e.g., heat, pepsin, and trypsin treatments) also face challenges, as these methods require purified toxins and controlled experimental conditions, limiting their applicability to real-world food samples with variable contaminant levels [48].

Finally, biochemical methods generally lag behind molecular techniques in speed and precision. Manual processes, such as culturing on selective media or enzymatic assays, prolong turnaround times compared to PCR or mass spectrometry. Additionally, their dependency on phenotypic expression makes them vulnerable to strain variability or atypical biochemical profiles, reducing diagnostic accuracy in outbreaks involving emerging or resistant strains [36,37].

## 5. Mass Spectrometry-Based Methods

### 5.1. Objective and Methodology

The primary objectives of MALDI-TOF MS in detecting *S. aureus* are to achieve rapid and accurate species identification in foodborne outbreak investigations, particularly in complex food matrices such as raw and processed milk, meat, and pastry samples. This method ensures reliable species-level identification compared to classical biochemical approaches, addressing the need for precision in epidemiological studies [24,49]. Additionally, the technique aims to differentiate MRSA from MSSA by analyzing protein spectral differences, facilitating targeted antimicrobial resistance profiling. Finally, MALDI-TOF MS is designed to streamline high-throughput bacterial identification in clinical and food safety laboratories, replacing labor-intensive methods such as biochemical testing or 16S rRNA sequencing with a standardized and automated workflow [45].

The MALDI-TOF MS methodology involves a systematic workflow beginning with isolating and culturing bacterial colonies from food samples (e.g., milk, meat) or clinical specimens using selective agar media. Pure colonies are cultured under standardized conditions (e.g., 37 °C for 18–24 h) to ensure viability and homogeneity [45]. Following this, protein extraction is performed by suspending a single colony in 70% formic acid to lyse bacterial cell walls and solubilize intracellular proteins, including ribosomal proteins critical for generating spectral profiles. Acetonitrile may be added to precipitate impurities, enhancing spectral clarity [24,45].

The extracted proteins are then spotted onto a steel MALDI target plate (Billerica, USA), air-dried, and overlaid with a matrix solution to co-crystallize with proteins, facilitating efficient laser desorption and ionization. A nitrogen laser irradiates the sample, vaporizing and ionizing proteins into charged molecules, which are accelerated through a vacuum flight tube. The time-of-flight (TOF) of these ions is measured to generate mass-to-charge (*m*/*z*) spectra dominated by ribosomal proteins (4–20 kDa range), which serve as species-specific biomarkers [24].

Acquired spectra are algorithmically compared against a proprietary database containing reference profiles of *S. aureus*, MRSA, MSSA, and other Staphylococcal species. Spectral matches are validated using scoring systems (e.g., log-score values ≥ 2.0 indicate species-level identification), ensuring reliable strain differentiation. The results are cross-verified with clinical or food safety data, categorizing isolates as MRSA or MSSA based on spectral concordance with resistance markers [24,45,49].

### 5.2. Performance

MALDI-TOF MS demonstrates exceptional analytical performance in detecting and identifying *S. aureus*. Studies report sensitivity and specificity exceeding 99% when analyzing 44 Staphylococcal isolates, including MRSA and MSSA strains, with spectral matching enabling precise differentiation [24]. The method outperforms traditional biochemical techniques and 16S rRNA sequencing in speed, delivering results within hours compared to days, which is the length of time required for phenotypic assays or genetic sequencing [24,45]. Its high-throughput capability allows simultaneous analysis of multiple isolates, reducing operational costs and labor compared to manual biochemical testing [45].

In food safety applications, MALDI-TOF MS has been validated for direct use with raw and processed food samples, such as milk, meat, and pastry, achieving reliable species identification without extensive pre-analytical purification steps [49]. The method’s accuracy surpasses classical approaches, as evidenced by its ability to confirm *S. aureus* identity in complex matrices with minimal false positives or negatives [24,49]. However, performance metrics such as sensitivity or specificity in specific food matrices (e.g., dairy and meat) are not explicitly quantified in the sourced studies, and the requirement for isolated colonies may introduce delays in scenarios requiring rapid outbreak response.

### 5.3. Limitations/Challenges

The sourced studies explicitly reporting on MALDI-TOF MS for *S. aureus* detection did not address specific limitations or challenges of the method in the context of foodborne outbreak investigations. While MALDI-TOF MS is highlighted for its high sensitivity, specificity, and rapid turnaround time compared to traditional biochemical or molecular methods, the provided data lack a discussion of potential constraints [50]. For instance, the cited works do not mention general challenges associated with mass spectrometry, such as the necessity for isolated bacterial colonies before analysis, which may delay results in time-sensitive outbreak scenarios. Similarly, interference from complex food matrices, a common concern in food microbiology, is not quantitatively evaluated or reported in the studies. The absence of explicit performance metrics (e.g., sensitivity and specificity) in specific food matrices (e.g., dairy and meat) further limits the assessment of practical applicability in diverse food safety contexts.

## 6. Immunological Methods for Detecting *Staphylococcus aureus* and Its Enterotoxins in Foodborne Intoxication Epidemics

### 6.1. Objective and Methodology

The immunological detection of *S. aureus* SEs in foodborne outbreaks is driven by objectives tailored to specific operational needs, including rapid on-site screening, precise quantification, and simultaneous identification of multiple toxin serotypes. Methodologies vary in complexity and application, leveraging antibody specificity and diverse detection platforms to address challenges posed by food matrices [51].

Sandwich ELISA techniques are central for their quantitative precision and adaptability. For example, Sandwich ELISA targeting *S. aureus* Protein A has been developed, using a 24 h incubation in selective broth followed by detection on an ELISA plate coated with anti-Protein A antibodies. This test can detect *S. aureus* at levels as low as <2 CFU/g in processed and precooked foods within 28 h, providing a faster alternative to traditional culture techniques [52].

In addition, a specialized Sandwich ELISA for SEG detection employs IgY chicken antibodies to capture SEG, effectively avoiding interference from SpA in complex matrices like milk. Detection is achieved using rabbit anti-SEG antibodies conjugated to horseradish peroxidase, enabling sensitivity as low as 1 ng/mL in spiked and natural food samples [53,54]. Similarly, a Sandwich ELISA targeting SEQ utilizes monoclonal antibodies to quantify SEQ in *S. aureus* culture supernatants, with a linear detection range of 0.5–20 ng/mL and no cross-reactivity with other enterotoxins [48]. These protocols often involve 96-well microtiter plates, standardized toxin controls, and enzymatic signal amplification to ensure reproducibility.

Automated systems like VIDAS^®^ SET2 (France) integrate enzyme-linked fluorescent assays (ELFA) to streamline SE detection in food samples. Cheese, tuna, and other matrices are homogenized, pH-adjusted, and centrifuged before automated analysis, minimizing manual intervention and enhancing throughput during outbreak investigations. This system detects classical SEs (SEA–SEE) through fluorescently labeled antibodies, with results generated via optical analysis [55,56].

Multiplex immunochromatographic tests address the need for simultaneous toxin detection. For instance, a pre-industrial lateral flow assay incorporates antibody pairs for SEA, SEB, SEG, SEH, and SEI on a single strip, enabling on-site identification within 30 min. While effective in buffer-based controls, sensitivity declines in complex samples like human feces or raw vegetables due to matrix interference. Simpler monoplex immunochromatographic tests for SEG, SEH, and SEI use gold nanoparticle-conjugated antibodies, providing rapid visual results for raw foods (e.g., shrimp and chicken) and clinical diarrhea samples, albeit with reduced sensitivity in challenging matrices [6].

Additional immunochromatographic tests have been developed for *S. aureus* detection in foods, offering rapid and simple on-site screening through specific antibody pairs [57].

Reversed passive latex agglutination (RPLA) methods, such as the SET-RPLA kit (New York, NY, USA), offer semi-quantitative detection of classical SEs (SEA–SED) through visible agglutination in culture supernatants or food extracts. This cost-effective approach bypasses the need for sophisticated equipment, making it suitable for resource-limited settings, although it lacks the quantitative rigor of ELISA [58,59].

The European Screening Method (ESM) enhances sensitivity in low-concentration samples by combining protein extraction and dialysis to concentrate SEs from complex food matrices like dairy products. This preparatory step is critical for downstream immunological assays, ensuring toxins are detectable even at trace levels [60].

### 6.2. Performance

Immunological methods for detecting *S. aureus* enterotoxins exhibit distinct performance profiles, shaped by their design, operational context, and the challenges posed by food matrices. ELISAs, particularly monoclonal antibody-based formats, demonstrate superior sensitivity and quantification capabilities. For example, Sandwich ELISAs targeting SEG and SEI achieve detection limits as low as 0.2–0.3 ng/mL, outperforming polyclonal assays that require higher thresholds (e.g., 1 ng/mL for SEG) [6]. Quantitative ELISAs excel in precision, detecting SEA and SED at 0.49 ng/g and 2.04 ng/g, respectively, in complex food samples like home-canned tuna [55]. However, these assays demand extended processing times (several hours), limiting their utility in time-sensitive outbreak investigations.

In contrast, lateral flow immunochromatographic tests prioritize rapidity, delivering results within 30 min, but at the cost of reduced sensitivity in complex matrices. Monoplex assays detect SEG, SEH, and SEI at 0.01–0.1 ng/mL in buffer; yet, sensitivity declines threefold in diarrheal samples or raw vegetables due to nonspecific binding [6]. Multiplex variants, capable of detecting five toxins simultaneously, exhibit further sensitivity reductions (0.1–0.3 ng/mL), underscoring a trade-off between multiplexing capability and detection limits [6]. Despite these limitations, lateral flow tests remain indispensable for on-site screening, offering specificity rates of 0.95–1.0 and eliminating the need for specialized equipment.

Reversed passive latex agglutination (RPLA) methods, such as SET-RPLA, provide a cost-effective alternative for semi-quantitative detection of classical SEs (SEA–SED). These assays are simpler and faster than PCR, requiring minimal technical expertise, but lack quantitative precision and often fail to detect toxins below 1 ng/mL [58,59]. Their qualitative nature necessitates confirmatory testing, limiting their role in settings requiring precise toxin quantification.

Automated systems like VIDAS^®^ SET2 (France) and RIDASCREEN^®^ SET (Germany) bridge the gap between sensitivity and throughput. VIDAS^®^ SET2 (France), employing enzyme-linked fluorescent assays (ELFAs), automates sample processing and detects SEA–SEE with high specificity (1.0) in dairy products and tuna [56]. However, its sensitivity is inconsistent; it fails to detect SEA at 0.019 ng/g in food leftovers, highlighting vulnerabilities in low-concentration scenarios [1]. RIDASCREEN^®^ SET (Germany), while robust for quantifying multiple toxins in outbreak-related foods, shares similar limitations in complex matrices, underscoring the persistent challenge of matrix interference across automated platforms [1].

The choice of method hinges on context: ELISAs dominate in laboratories prioritizing precision, lateral flow tests excel in field deployments, and RPLA serves resource-limited environments. Automated systems, although efficient, remain constrained by cost and infrastructure demands. Across all methods, inconsistent reporting of sensitivity and specificity metrics—particularly for RPLA and older ELISA variants—complicates cross-method evaluations, emphasizing the need for standardized reporting in future studies [37,61].

### 6.3. Limitations and Challenges

Immunological methods for detecting SEs face several critical limitations, many of which stem from the inherent complexity of food matrices and methodological constraints. Matrix interference remains a pervasive challenge, particularly in heterogeneous samples such as cheese, raw vegetables, or diarrheal specimens. For instance, immunochromatographic tests exhibit a threefold reduction in sensitivity when analyzing complex matrices like human diarrhea compared to buffer-based controls, likely due to nonspecific protein binding or particulate matter [6]. Similarly, the VIDAS^®^ SET3 (France) assay demonstrates variable performance, detecting SEG and SEH at 0.2 ng/mL but requiring 0.5 ng/mL for SEA in food matrices, underscoring toxin- and matrix-dependent variability [6]. Even advanced methods like ESM require preparatory steps such as protein extraction and dialysis to concentrate toxins, highlighting the difficulty of detecting low-abundance SEs in unprocessed samples [60].

Sensitivity and specificity gaps further limit reliability. While monoclonal antibody-based ELISAs achieve high sensitivity (e.g., 0.2 ng/mL for SEG/SEI), automated systems like VIDAS^®^ SET2 fail to detect SEA at 0.019 ng/g in food leftovers, emphasizing inconsistencies in low-concentration scenarios [1]. Immunochromatographic tests, although rapid, show serotype-dependent sensitivity, with SEA detection at 0.3 ng/mL compared to 0.01 ng/mL for SEG [6]. Although generally robust, specificity is not universally reported; for example, latex agglutination methods (e.g., RPLA) often lack specificity metrics, complicating their validation [37]. Cross-reactivity risks persist in polyclonal antibody-based assays, necessitating innovations like IgY antibodies in SEG-specific ELISAs to avoid interference from SpA [53].

Qualitative and semi-quantitative limitations restrict the utility of several methods. RPLA and lateral flow assays provide rapid results but yield qualitative or semi-quantitative data, requiring confirmation through quantitative techniques like ELISA or PCR [59,62]. For example, SET-RPLA kits, while cost-effective, fail to quantify toxins below 1 ng/mL and produce false negatives in outbreak-related food samples, underscoring their inadequacy for precise toxin profiling [62].

Operational and technical barriers also hinder widespread adoption. Automated systems such as VIDAS^®^ SET2 demand specialized equipment and technical expertise, limiting their use in resource-limited settings [56]. Additionally, the subjective interpretation of results, such as visual assessment of agglutination in RPLA or band intensity in lateral flow tests, introduces variability, particularly in field deployments with untrained personnel [37].

Finally, inconsistent reporting of performance metrics obscures method comparisons. Many studies omit sensitivity or specificity data, particularly for older ELISAs and latex agglutination kits, complicating evidence-based method selection [61]. Furthermore, the focus of many assays on classical SEs (SEA–SEE) neglects emerging *egc* toxins (SEG, SEH, and SEI), creating gaps in detecting newer variants implicated in modern outbreaks [6,48]. Addressing these challenges requires standardized reporting protocols, improved antibody specificity, and innovations to mitigate matrix effects without sacrificing scalability.

## 7. PCR-Based Methods

### 7.1. Objective and Methodology

The primary objectives of PCR-based methods in detecting *S. aureus* during foodborne outbreaks encompass rapid pathogen identification, the differentiation of MRSA, and comprehensive profiling of virulence and antibiotic resistance genes. These techniques aim to address the limitations of conventional culture-based approaches by delivering faster and more specific results, particularly in complex food matrices such as dairy products, meats, and ready-to-eat foods [35,63].

Methodologically, the process begins with DNA extraction from diverse samples, including bacterial colonies, food matrices, and environmental swabs. Protocols vary, with studies employing commercial kits (e.g., Magen Biotech (China)) or simplified boiling methods to isolate bacterial DNA, ensuring purity and compatibility for downstream amplification [40,44]. Primer design is meticulously tailored to target specific genetic markers: *mecA* and *mecC* for methicillin resistance, *nuc* and *coa* for species-specific confirmation, and *sea-see* for enterotoxin gene detection [43,64]. Multiplex PCR enhances diagnostic efficiency by enabling simultaneous amplification of multiple targets, such as SCC*mec* types (I–VI) alongside virulence factors like toxic shock syndrome toxin (*tsst-1*) or Panton–Valentine leukocidin (*pvl*), facilitating holistic strain characterization in a single reaction [49,65].

Thermal cycling protocols typically follow standardized conditions, including denaturation at 95 °C, annealing at 56 °C, and extension at 72 °C, with subsequent analysis via agarose gel electrophoresis to visualize amplicons of expected sizes [35,66]. Real-time PCR (qPCR) further refines detection by monitoring fluorescence in real time, utilizing SYBR Green (WA, USA) or TaqMan^®^ (Foster, USA) probes to quantify target genes such as *egc* clusters or *pvl*, thereby improving sensitivity for low-abundance genetic material [67,68]. For validation, the sequencing of PCR products or comparison with control strains is employed, particularly in studies investigating genetic diversity or novel gene variants [39,65].

Advanced methodologies incorporate one-step PCR protocols optimized for rapid detection of up to eight toxin genes, significantly reducing processing time while maintaining specificity [67]. Comparative analyses with traditional methods, such as phenotypic antibiotic susceptibility testing or culture-based enterotoxin assays, are routinely conducted to validate PCR’s superior sensitivity and specificity [69,70]. However, challenges persist in standardizing protocols across diverse food matrices, as inhibitors in dairy or meat products may impede DNA amplification efficiency [43,71].

### 7.2. Performance

PCR-based methods demonstrate high sensitivity and specificity in detecting *S. aureus* and its associated markers in foodborne outbreaks. For methicillin resistance detection, amplifying the *mecA* gene achieved 100% sensitivity and specificity in confirming MRSA strains across multiple studies, aligning perfectly with phenotypic resistance profiles [35,72]. Similarly, species-specific identification via the *nuc* gene exhibited 100% sensitivity in differentiating *S. aureus* from other *Staphylococci*, as validated in poultry and dairy samples. Enterotoxin gene detection, however, showed variability depending on the target: *hla* and *hld* genes were detected with 94.5% sensitivity in chicken livers, while *sea* gene sensitivity dropped to 80% in food isolates [66,73]. qPCR further enhanced precision, identifying low-prevalence virulence factors like *pvl* (11.1%) and *tsst-1* (3.2%) in foodborne isolates, which are often missed by conventional methods [49].

PCR consistently outperformed traditional culture-based assays in speed and resolution. For instance, multiplex PCR detected SCC*mec* types IVa, II, and III in MRSA strains with higher accuracy than biochemical typing, enabling precise epidemiological tracking [43,65]. In enterotoxin profiling, PCR identified toxin genes in 55% of isolates, surpassing the lower yields of phenotypic methods, and revealed higher prevalence of *seg* (23.8%) and *sec* (28.6%) in dairy and meat samples [70,74]. Multiplex protocols proved particularly robust, achieving 92.3% positivity for toxin genes in one-step reactions without nonspecific amplification, as demonstrated in fermented foods and ready-to-eat samples [67,75].

Despite these advantages, limitations persist. Sensitivity metrics for certain targets, such as *SEj* (16.6%) and *SEr* (14.3%), remained suboptimal, highlighting variability in primer efficiency [76]. Additionally, PCR’s inability to confirm active toxin production—merely detecting gene presence—necessitates complementary methods like ELISA for functional validation [6]. Food matrix interference further complicates DNA extraction in dairy and meat products, requiring protocol optimization to mitigate inhibition [44,71]. Finally, inconsistent sensitivity and specificity data reporting in studies targeting toxin genes or SCC*mec* subtypes underscores the need for standardized validation frameworks [36,63].

### 7.3. Limitations/Challenges

PCR-based methods, while powerful, face several challenges in detecting *S. aureus* during foodborne outbreaks. A key limitation is the subjectivity inherent in gel electrophoresis, which relies on the visual interpretation of amplicon bands. This introduces variability, particularly when distinguishing closely sized products or confirming faint bands, as noted in studies analyzing SCC*mec* types or multiplex enterotoxin gene panels [39,77]. Additionally, food matrix interference complicates DNA extraction and amplification efficiency. Inhibitory compounds in dairy, meat, and ready-to-eat foods often reduce PCR sensitivity, necessitating protocol adjustments such as dilution steps or alternative extraction kits [44,71]. For example, dairy matrices like cheese and milk require specialized DNA purification to mitigate inhibition, as observed in studies targeting *coa* or *mecA* genes [44,72].

Another critical challenge is the disconnection between gene presence and toxin production. PCR detects genetic markers but does not confirm active toxin expression, which is essential for assessing pathogenicity. Studies detecting *seg* or *sei* genes in *S. aureus* isolates, for instance, could not correlate these findings with functional enterotoxin activity without supplementary methods like ELISA [6,54]. Furthermore, variability in primer efficiency impacts detection rates, as seen with low sensitivity for *SEj* (16.6%) and *SEr* (14.3%) in fresh meat and quick-frozen food samples, highlighting the need for optimized primer design [76].

Inconsistent reporting of performance metrics across studies further complicates method validation. Many investigations on SCC*mec* typing, toxin gene profiling, or *agr* locus analysis omitted sensitivity or specificity data, limiting reproducibility and cross-study comparisons [36,63]. For instance, while *mecA* detection consistently achieved 100% sensitivity, studies targeting *pvl* or *tsst-1* often lacked specificity metrics, hindering comprehensive risk assessments [49,68].

Finally, resource and technical demands restrict field applicability. qPCR, although precise, requires expensive equipment and reagents, as seen in SYBR Green-based assays for *egc* clusters or TaqMan^®^ kits (WA, USA) for *sea* detection [55,67]. Similarly, multiplex protocols for the simultaneous detection of 18 toxin genes demand rigorous optimization to avoid nonspecific amplification, limiting their use in resource-constrained settings [61,75]. These challenges underscore the need for standardized protocols, improved primer validation, and complementary assays to bridge gaps between genetic detection and functional pathogenicity.

## 8. Isothermal Amplification-Based Methods

### 8.1. Objective and Methodology

Isothermal amplification methods are tailored to address specific diagnostic needs in detecting *S. aureus* during foodborne outbreaks. CPA aims to rapidly identify MRSA toxins (*sea*, *seb*, and *pvl*) in clinical and foodborne strains [78]. This method employs primers specific to toxin genes, with amplification conducted at 63 °C for 60 min using strand-displacing polymerases. Detection relies on turbidity from magnesium pyrophosphate precipitation or fluorescence from DNA-binding dyes, validated across MRSA, MSSA, and non-*Staphylococci* strains.

Multi-LAMP (loop-mediated isothermal amplification) focuses on simultaneously detecting *S. aureus* in food samples [79]. Primers targeting *nuc* enable amplification at a constant temperature for 40 min, utilizing six primers per target to drive auto-cycling strand displacement. The results are visualized via agarose gel electrophoresis or real-time fluorescence, achieving 100% sensitivity and specificity in inclusivity/exclusivity panels.

For food matrices, m-LAMP/LFA (multiplex LAMP with lateral flow assay) targets SEA- and SEB-producing *S. aureus* in milk, apple juice, cheese, and rice [80]. DNA extracted from samples undergoes 30 min amplification at 63 °C for *sea* and *seb* genes, followed by lateral flow detection using biotin- and FITC-labeled amplicons. Gold nanoparticle probes on LFA strips provide visual confirmation within 30 min, with specificity confirmed by non-reactivity to non-target pathogens.

Multiplex LAMP extends concurrent detection to *nuc* genes in food samples, optimizing primer compatibility for single-reaction amplification. Amplification at a constant temperature is monitored via agarose gel electrophoresis, validated using pure cultures and spiked food samples to achieve 100% accuracy [79].

Lastly, PMA-CPA (Propidium Monoazide Combined with CPA) selectively identifies viable but non-culturable (VBNC) MRSA cells in food (e.g., Cantonese cake) and clinical isolates [78]. PMA pretreatment crosslinks DNA from dead cells under light, ensuring only viable-cell DNA is amplified via CPA targeting *sea*, *seb*, and *pvl* genes at 63 °C for 60 min. Specificity for VBNC cells is confirmed through alignment with conventional culture methods.

### 8.2. Performance

Isothermal amplification methods demonstrate distinct performance metrics in detecting *S. aureus* and its toxins, with notable advantages over conventional PCR. CPA exhibited sensitivities of 75 ng/µL, 107.5 ng/µL, and 85 ng/µL for *sea*, *seb*, and *pvl* toxin genes, respectively, outperforming traditional PCR in sensitivity for MRSA toxin detection. However, specificity data were not reported, limiting comprehensive validation [78]. Multi-LAMP achieved 100% sensitivity and specificity in inclusivity/exclusivity testing, detecting *S. aureus* with a 10-fold higher sensitivity than PCR and a rapid 40 min runtime [79].

For food matrices, m-LAMP/LFA demonstrated a visual limit of detection (LOD) of 10^2^ CFU/mL for *sea*- and *seb*-producing *S. aureus*, with 10-fold greater sensitivity than PCR and results obtainable within 30 min. Specificity was confirmed by the absence of cross-reactivity with non-target pathogens, making it suitable for complex food samples [80]. Similarly, multiplex LAMP reported 100% sensitivity and specificity (denoted as 1) for concurrent *nuc* gene detection, surpassing PCR sensitivity by 10-fold [79].

PMA-CPA addressed viability detection challenges, showing 100% specificity for viable but non-culturable (VBNC) MRSA cells in food and clinical samples. While sensitivity metrics were unreported, its results aligned fully with conventional VBNC confirmation methods, with significantly faster turnaround times [78].

Comparative analyses highlight consistent trends: isothermal methods reduce detection times by 50–75% (30–60 min vs. 2–4 h for PCR) and enhance sensitivity by 10-fold, critical for rapid outbreak response. However, gaps in specificity (CPA) or sensitivity (PMA-CPA) reporting underscore the need for standardized validation protocols. Despite these limitations, the methods’ ability to bypass thermocyclers and deliver field-compatible results positions them as transformative tools for foodborne pathogen surveillance [78,79,80].

### 8.3. Limitations/Challenges

Despite their advantages, isothermal amplification methods face several constraints in practical applications for detecting *S. aureus* in foodborne outbreaks. Subjectivity in result interpretation arises in methods relying on visual readouts, such as turbidity measurements in CPA or lateral flow assay (LFA) strips in m-LAMP/LFA, which may introduce variability in data interpretation [78,80]. For instance, m-LAMP/LFA’s visual detection limit (10^2^ CFU/mL) depends on the operator’s discernment of colorimetric signals, potentially affecting reproducibility in resource-limited settings.

Incomplete performance metrics limit comparative analyses. While demonstrating sensitivities of 75–107.5 ng/µL for MRSA toxins, CPA lacks specificity data, hindering comprehensive validation. Similarly, PMA-CPA reports 100% specificity for VBNC MRSA cells but omits sensitivity values, complicating its reliability assessment [78]. Although tested in milk, cheese, and apple juice, food matrix interference was not explicitly evaluated in these studies, leaving uncertainties about inhibitors (e.g., fats and proteins) affecting amplification efficiency [80].

Technical complexity is another challenge. Multi-LAMP and multiplex LAMP require meticulous primer design for simultaneous pathogen detection, increasing the risk of nonspecific amplification or primer dimerization [79]. While agarose gel electrophoresis validates results, it is labor-intensive and less suited for quantitative analysis, limiting high-throughput applications. Additionally, methods like PMA-CPA involve multi-step workflows (e.g., PMA pretreatment and amplification), which may offset time-saving benefits in field settings [78].

Finally, standardization gaps persist. Variations in incubation times (30–60 min) and temperatures (63 °C, unspecified in some protocols) across studies challenge reproducibility [79,80]. Despite these limitations, isothermal methods remain promising, provided future work addresses these hurdles through rigorous validation and protocol harmonization.

## 9. Sequencing-Based Methods

### 9.1. Objective and Methodology

Sequencing-based methods are primarily employed to genetically characterize *S. aureus* strains, investigate outbreaks, and profile antimicrobial resistance (AMR) and virulence determinants. WGS serves as a comprehensive approach for genotypic analysis, aiming to decode the entire genome of *S. aureus* isolates [81]. For example, WGS has been applied to 39 representative *S. aureus* strains from raw pork samples, utilizing the Illumina MiSeq (San Diago, CA, USA) platform for sequencing, followed by bioinformatic tools such as SPAdes and Quast to assemble genomes and identify resistance and virulence genes [63]. Similarly, in another study, DNA extracted from ready-to-eat food isolates was sequenced using Illumina MiSeq, with pan-genome and phylogenomic analyses conducted to compare genomic data against global databases, thereby identifying strain-specific markers linked to virulence and resistance [82]. These methodologies enable detailed insights into genetic diversity, toxin gene distribution, and phylogenetic relationships, supporting outbreak surveillance and source tracking [45,83].

MLST focuses on strain typing and epidemiological analysis by sequencing seven housekeeping genes [84]. For instance, MRSA isolates from table eggs underwent DNA extraction and PCR amplification of loci such as *arcC* and *gmk*, followed by the sequencing and assignment of STs via the *S. aureus* MLST database. This approach identified dominant lineages like ST772 and ST8, critical for understanding clonal spread in foodborne outbreaks [35]. In another study, MLST was applied to 250 *S. aureus* isolates, sequencing seven housekeeping genes and analyzing sequence types to trace the origin of staphylococcal enterotoxin (SE) genes, demonstrating higher discriminatory power compared to *spa* typing [77]. Clonal complex analysis using eBURST (UK) or BioNumerics software (Belgium) further revealed genetic clusters, such as CC45 in isolates from leftover food items, linking strains to specific outbreak sources [62,85].

Targeted sequencing methodologies address specific objectives, such as confirming the presence of virulence genes or identifying genetic variants. For example, the *seh* gene, associated with enterotoxin H production, was amplified via PCR and sequenced in isolates from the Pitești outbreak, revealing 100% identity with reference sequences and confirming its role in intoxication [56]. Similarly, Sanger sequencing of promoter regions and coding sequences of *seb*, *sec*, and *sed* genes in diverse *S. aureus* strains uncovered novel nucleotide variants, elucidating genetic diversity linked to toxin production [6].

Broad-spectrum pathogen detection is achieved through 16S rRNA amplicon sequencing, which targets hypervariable regions (V3–V5) of the 16S gene. This method employed the Illumina MiSeq platform to sequence amplicons in fermented foods, with data analyzed using QIIME and MG-RAST. This approach detected additional pathogens like *B. cereus* and *P. mirabilis* alongside *S. aureus*, offering a more inclusive view of microbial contamination than qPCR [86].

### 9.2. Performance

The performance of sequencing-based methods in detecting *S. aureus* during foodborne outbreaks is primarily evaluated through their resolution, discriminatory power, and ability to provide comprehensive genetic insights. However, the studies did not explicitly report sensitivity and specificity metrics. WGS performs more in genetic profiling than traditional methods, such as PCR or PFGE. For instance, WGS enabled the identification of complete toxin repertoires and antimicrobial resistance genes across diverse *S. aureus* strains, offering a level of genomic detail unattainable with conventional techniques [63,87]. In one study, WGS of 39 *S. aureus* strains from raw pork samples revealed strain-specific virulence factors and resistance markers, facilitating precise outbreak tracking [63]. Similarly, pan-genome analyses using WGS data linked foodborne isolates to global strain databases, enhancing epidemiological insights [82].

MLST outperforms traditional typing methods in genetic discrimination. For example, MLST analysis of 250 *S. aureus* isolates provided higher resolution than *spa* typing for tracing the origin of SE genes, which is crucial for identifying outbreak sources [77]. This method also effectively clustered strains into clonal complexes, such as CC45 in isolates from leftover food items, clarifying transmission pathways during outbreaks [62]. Additionally, MLST identified novel sequence types (e.g., ST72 in imported meats) and livestock-associated lineages (e.g., ST398), underscoring its utility in mapping genetic diversity [34,65].

Targeted sequencing methods, such as *seh* gene analysis, achieved 100% identity with reference sequences in outbreak isolates, confirming gene presence and stability [56]. Similarly, Sanger sequencing of *seb*, *sec*, and *sed* promoter regions uncovered novel nucleotide variants, highlighting genetic diversity linked to toxin production [6]. These approaches provide precise gene-level resolution but lack comparative sensitivity or specificity data against traditional assays.

Broad-spectrum methods like 16S rRNA amplicon sequencing (MiSeq) demonstrated enhanced pathogen detection capabilities. This method identified additional pathogens such as *B. cereus* and *P. mirabilis* alongside *S. aureus* in fermented foods, outperforming qPCR in inclusivity [86]. While these sequencing-based methods excel in resolution and comprehensiveness, their performance is constrained by resource intensity, reliance on bioinformatics expertise, and the absence of standardized sensitivity or specificity metrics in the reviewed studies.

### 9.3. Limitations/Challenges

A significant limitation of sequencing-based methods is the absence of reported sensitivity and specificity data across studies, which hinders direct comparisons with traditional techniques like PCR or serotyping [35,61,63]. WGS, while offering unparalleled genomic resolution, is notably resource-intensive, requiring advanced bioinformatics infrastructure and expertise for data analysis and interpretation, as highlighted in studies characterizing foodborne isolates [82,83]. This resource demand limits its accessibility for routine use in some settings.

MLST, although effective for epidemiological tracking, depends heavily on standardized databases for sequence type assignments, and its resolution may falter when distinguishing closely related strains, as noted in analyses of MRSA isolates [88]. Furthermore, inconsistencies in performance comparisons with other typing methods, such as the lack of direct benchmarks against PFGE or MLVA in certain studies, complicate the evaluation of its relative advantages [88,89]. Variability in bioinformatics pipelines—such as the use of SPAdes (Russia), Quast (Germany), eBURST (United Kingdom), or BioNumerics (Belgium) across studies—introduces potential subjectivity in data interpretation, as divergent tools may yield inconsistent phylogenetic or clonal complex classifications [63,85]. Additionally, precise targeted gene sequencing approaches lack comparative data on detection limits or specificity against conventional assays, limiting their validation in complex food matrices [6,56]. These challenges underscore the need for standardized protocols, enhanced reporting of performance metrics, and improved accessibility to computational resources to maximize the utility of sequencing-based methods in foodborne outbreak investigations.

## 10. Sequence-Based Typing Methods

### 10.1. Objective and Methodology

The primary objectives of sequence-based spa typing in foodborne *S. aureus* investigations encompass strain differentiation, genetic characterization, identification of clonal relationships, and epidemiological tracking of outbreaks. Spa typing achieves strain differentiation by analyzing polymorphisms in the variable repeat regions of the *spa* gene, enabling precise discrimination between isolates [90]. For example, distinct spa types such as t657, t8645, and t13252 were identified to differentiate MRSA lineages or trace clonal origins in food matrices like table eggs, milk, and pork [35,55,77]. This method also facilitates genetic characterization, with one study resolving 103 *spa* types among 568 isolates, highlighting its capacity to uncover genetic diversity [77].

Clonal distribution analysis is another critical objective, linking *spa* types to STs to map strain evolution and transmission pathways. For instance, *spa* type t657 correlated with ST772, while t8645 aligned with ST8, demonstrating the method’s utility in understanding clonal dynamics within food production chains [35,91]. Epidemiological tracking associates specific *spa* types, such as t701, t002, and t172, with contamination sources like bulk tank milk or environmental swabs, thereby identifying transmission routes during outbreaks [38,91].

Methodologically, *spa* typing follows a standardized workflow. The polymorphic X region of the *spa* gene is first amplified via PCR using conserved primers, targeting hypervariable repeats flanked by stable sequences [33,92]. Amplified products are then sequenced to resolve repeat patterns, with protocols tailored to diverse samples, including MRSA isolates from pigs, dairy products, and chicken livers [88,93]. Bioinformatics tools such as Ridom SeqSphere+ (Germany), BioNumerics (Belgium), and SpaServer (Germany) compare sequences against global databases to assign standardized spa types, ensuring reproducibility and cross-study comparability [40,77,94]. Data interpretation involves analyzing repeat arrangements to infer genetic relatedness, often visualized through minimum spanning trees or clustering algorithms [70,91].

This methodology has been applied to diverse food matrices, including milk, eggs, pork, and environmental swabs, demonstrating its adaptability to complex sample types. For example, spa typing of chicken liver isolates revealed high genetic diversity linked to human-associated sources, underscoring its utility in tracing contamination origins [94]. Similarly, studies on bulk tank milk and dairy products employed the technique to characterize MRSA strains, reinforcing its role in food safety surveillance [46,72].

### 10.2. Performance

The performance of sequence-based *spa* typing in detecting and characterizing *S. aureus* in foodborne outbreaks is characterized by its high discriminatory power. However, quantitative metrics such as sensitivity and specificity were rarely reported across studies. Most investigations did not provide numerical sensitivity or specificity values, limiting direct comparisons with traditional methods [33,38,41,70,77,92]. Despite this, spa typing demonstrated robust strain differentiation, resolving up to 103 distinct *spa* types in a large-scale analysis of 568 isolates, with 18 primary types dominating the dataset [77]. This high resolution enabled the identification of novel *spa* types, such as t13252, which was uniquely associated with epidemiological tracking of isolates [55].

Comparative analyses highlighted *spa* typing’s utility in outbreak investigations, particularly in correlating genetic diversity with contamination sources. For example, chicken liver isolates exhibited high *spa* type diversity, with many linked to human-associated origins, underscoring the method’s ability to trace transmission routes [94]. Similarly, *spa* typing correlated well with MLST in studies of pasteurized milk isolates, supporting its reliability for epidemiological studies [40]. However, discrepancies between *spa* types and MLST-based clonal classifications were noted. For instance, strains sharing the same ST exhibited divergent *spa* types, suggesting potential limitations in phylogenetic resolution [85].

Performance evaluations against traditional methods were limited. One study reported *spa* typing’s high-resolution genotyping compared to older techniques but did not quantify superiority [41]. Another investigation of MRSA isolates from pigs and workers highlighted *spa* typing’s ability to reveal genetic diversity but omitted direct comparisons with conventional methods, complicating assessments of its relative advantages [88]. Despite these gaps, the method’s reproducibility and adaptability to diverse matrices—including milk, eggs, and environmental samples—were consistently emphasized, with studies successfully applying it to characterize strain lineages and track clonal distributions [46,72,91].

### 10.3. Limitations/Challenges

Despite its utility, sequence-based spa typing exhibits several limitations in foodborne *S. aureus* investigations. A significant challenge is the widespread absence of reported sensitivity and specificity metrics across studies, hindering quantitative assessments of the method’s accuracy and reliability compared to traditional approaches. For instance, multiple investigations explicitly stated that sensitivity and specificity values were “Not Reported” or “Not Applicable,” limiting objective evaluations of performance [33,38,41,46,70,92]. This gap complicates efforts to standardize the method for regulatory or diagnostic applications in food safety.

Another limitation arises from discrepancies between *spa* typing and MLST. While *spa* types often correlate with STs, studies noted instances where strains sharing the same ST exhibited divergent spa types, reducing their reliability for inferring deep phylogenetic relationships. For example, *spa* typing did not consistently align with MLST-based clonal classifications, suggesting potential limitations in resolving broader evolutionary lineages [40,85]. Additionally, the method’s reliance on a single genetic locus (the *spa* gene) may overlook genomic diversity captured by whole-genome sequencing or multi-locus approaches, potentially underestimating strain complexity.

Comparative evaluations of *spa* typing against conventional methods, such as PFGE or phage typing, were notably scarce. Several studies highlighted its high discriminatory power but omitted direct comparisons with older techniques, leaving its relative resolution, cost, or speed advantages unclear [41,88,91]. Furthermore, the lack of standardized protocols for data interpretation, such as variability in bioinformatics tools (e.g., Ridom SeqSphere+ (Germany), BioNumerics (Belgium), and SpaServer (Germany)), could introduce inconsistencies in *spa* type assignments across laboratories, affecting reproducibility in multi-center studies [77,94].

Lastly, while *spa* typing is adaptable to diverse food matrices, its application to low-biomass or highly contaminated samples (e.g., environmental swabs and complex food products) may require optimized DNA extraction and amplification protocols to mitigate potential PCR inhibitors or background noise. However, such technical challenges were not explicitly addressed in the reviewed studies.

## 11. Pulsed-Field Gel Electrophoresis-Based Methods

### 11.1. Objective and Methodology

The primary objective of PFGE in foodborne intoxication studies is to achieve high-resolution molecular typing of *S. aureus* strains for outbreak tracing, clonal dissemination analysis, and epidemiological surveillance [95,96]. This method is employed to discern genetic relatedness between isolates from diverse sources, including food matrices (e.g., dairy and table eggs), clinical specimens, and environmental samples, thereby identifying transmission pathways or contamination sources [62,66,74]. For instance, studies targeting MRSA strains utilized PFGE to differentiate human-acquired and livestock-associated lineages, linking specific pulsotypes (e.g., USA300 and USA500) to their origins [66,72].

Methodologically, PFGE protocols begin with the enzymatic digestion of bacterial genomic DNA using restriction endonucleases, predominantly SmaI, although XbaI and XmaI are also employed depending on the strain and study design [38,42]. The digested DNA is then embedded in agarose plugs and electrophoresed under standardized conditions, such as those outlined by PulseNet or CDC guidelines, using specialized systems like the CHEF (Contour-Clamped Homogeneous Electric Field) apparatus. Parameters such as pulse time, voltage, and run duration are optimized to resolve large DNA fragments [35,97]. Post-electrophoresis, gels are stained with DNA-binding dyes, and banding patterns are digitized for computational analysis. Software tools like BioNumerics (TX, USA) facilitate pattern comparison through clustering algorithms (e.g., Dice coefficient and UPGMA), enabling the classification of isolates into pulsotypes or composite types based on genetic similarity thresholds [56,65].

Studies involving complex food matrices (e.g., dairy products) emphasized rigorous DNA extraction protocols to mitigate interference from inhibitors, ensuring optimal restriction digestion and electrophoretic resolution [36,38]. For example, PFGE applied to *S. aureus* isolates from leaf vegetables, livestock, and humans incorporated PulseNet protocols to standardize fragment separation, followed by an integrative analysis of PFGE patterns, toxin genes, and antimicrobial resistance profiles [97]. In outbreak investigations, PFGE’s reproducibility allowed for a cross-comparison of pulsotypes across geographically dispersed isolates, confirming genetic diversity or clonal spread [60,64].

### 11.2. Performance

Sensitivity and specificity metrics for PFGE were not explicitly reported in the reviewed studies, reflecting a common gap in standardized quantitative assessments. However, the method’s performance was extensively evaluated through its discriminatory power and resolution in strain differentiation. PFGE demonstrated high genotypic discrimination, identifying 12 distinct genotypes among *S. aureus* isolates with a Simpson’s index of diversity of 0.909, indicative of robust resolution for outbreak investigations [65]. Similarly, another study detected 16 PFGE patterns in bovine milk isolates, suggesting clonal dissemination within populations [42]. The technique’s ability to link specific pulsotypes, such as USA300 and USA500, to human-acquired MRSA lineages further underscores its utility in molecular epidemiology [66].

Comparative analyses highlight PFGE’s superiority over traditional typing methods, enhancing strain differentiation through detailed banding patterns. For instance, PFGE profiles grouped isolates into major, intermediate, and unique pulsotypes in dairy and environmental samples, offering granular insights into genetic relatedness [74]. PFGE resolved 100% similarity between strains in outbreaks involving food and human sources, confirming clonal spread [60]. At the same time, other studies reported minimal diversity, such as only two genotypic patterns in MRSA isolates from table eggs [35]. This variability in performance underscores its context-dependent applicability, where factors like restriction enzyme choice and sample type influence outcomes.

PFGE was considered more time-consuming than PCR-based methods despite its high resolution, particularly in protocols requiring DNA digestion, electrophoretic separation, and computational analysis [72]. Nevertheless, its reproducibility and standardization (e.g., PulseNet protocols) enabled cross-laboratory comparisons, essential for multi-source outbreak tracing [97]. One study noted PFGE’s strong discriminatory power but observed no correlation between pulsotypes and *egc* toxin gene profiles, emphasizing the need for complementary methods in comprehensive strain characterization [89]. Overall, PFGE remains a cornerstone for clonal analysis, balancing high-resolution genotyping with operational complexities inherent to its methodology [56,64].

### 11.3. Limitations/Challenges

A significant limitation of PFGE lies in the subjectivity associated with interpreting DNA banding patterns, particularly when relying on visual inspection without robust computational tools. While software like BioNumerics aids in pattern analysis, inconsistencies in manual gel reading can lead to variability in pulsotype classification, affecting reproducibility across studies [64,89]. This challenge is compounded in studies involving complex food matrices, such as dairy products, fecal samples, or environmental swabs, where residual inhibitors from the sample matrix may interfere with DNA extraction or restriction enzyme efficiency, potentially skewing fragment resolution [38,74]. For instance, PFGE applied to *S. aureus* isolates from table eggs required stringent DNA purification protocols to mitigate matrix-derived interference butstill yielded minimal genotypic diversity, highlighting methodological constraints in certain contexts [35].

Another critical challenge is the absence of standardized sensitivity and specificity metrics across studies, as these parameters were consistently reported as “Not Reported” in the reviewed data. This gap limits comparative evaluations of PFGE’s performance relative to other typing methods and complicates the establishment of universal interpretive criteria [35,65]. Additionally, PFGE is notably time-intensive, requiring sequential steps—DNA digestion, prolonged electrophoretic runs (often exceeding 24 h), and computational analysis—making it less practical for rapid outbreak response compared to PCR-based alternatives [72].

Further limitations emerge from PFGE’s occasional inability to correlate with other genetic markers. For example, one study observed no association between PFGE pulsotypes and *egc* toxin gene profiles, underscoring the method’s restricted scope in capturing comprehensive strain characteristics without supplementary analyses [89]. Despite its high discriminatory power, PFGE may fail to resolve fine-scale genetic differences in clonal outbreaks, as evidenced by a 98.52% profile similarity in some *S. aureus* isolates, which could obscure minor genomic variations critical for precise outbreak tracing [64]. These limitations underscore the need to integrate PFGE with complementary molecular techniques to enhance epidemiological accuracy and address its inherent methodological constraints.

## 12. Other Molecular-Based Methods

### 12.1. NAuRA (Nice Automatic Research of Alleles)

#### 12.1.1. Objective and Methodology

NAuRA is designed to comprehensively identify all 27 enterotoxin genes in *S. aureus* strains isolated from food matrices and publicly available genome databases. The methodology begins with extracting genomic data and targeted screening for 26 enterotoxin genes using protein sequence alignment. BLAST-based homology searches facilitate the identification of conserved motifs, while subsequent allele-specific analysis distinguishes genetic variants. Phylogenetic trees are constructed to map evolutionary relationships, aiding in strain classification and outbreak tracking [87].

#### 12.1.2. Performance

NAuRA demonstrated a 93.7% concordance rate with conventional PCR methods when validated against a panel of *S. aureus* strains. However, sensitivity and specificity metrics were not explicitly reported, limiting direct comparisons with other quantitative detection platforms. The method’s reliance on bioinformatics tools ensures high-throughput capabilities but may introduce variability depending on database completeness [87].

#### 12.1.3. Limitations/Challenges

A key limitation of NAuRA is its dependence on pre-existing genome databases, which may omit novel or rare alleles not yet catalogued. Computational demands, including the need for advanced bioinformatics infrastructure, could hinder its application in resource-limited settings. Additionally, the absence of sensitivity and specificity data raises uncertainties about its reliability in complex food matrices, where inhibitory compounds might interfere with sequence accuracy. These factors underscore the necessity for supplementary validation when deploying NAuRA in epidemiological investigations [87].

### 12.2. CCB-Detection

#### 12.2.1. Objective and Methodology

The primary objective of CCB-Detection is to achieve ultra-sensitive detection of *S. aureus* in complex food matrices such as water, milk, juice, beer, and sour milk. The workflow begins with a simplified DNA extraction process from the sample, followed by PCR amplification of target sequences. Amplified DNA is then transcribed into RNA via in vitro transcription. The Cas13a enzyme, pre-complexed with a guide RNA specific to *S. aureus*, is introduced to the transcribed RNA. If the target RNA is present, Cas13a cleaves both the target and a quenched fluorescent reporter, producing a measurable signal [98].

#### 12.2.2. Performance

CCB-Detection achieved 100% sensitivity and specificity in validation studies, with a limit of detection (LOD) as low as 1 CFU/mL. This performance significantly surpasses traditional qPCR methods, which exhibit an LOD of 10^4^ CFU/mL under similar conditions. The method’s ability to bypass culturing steps and deliver results within hours highlights its advantage in rapid outbreak investigations [98].

#### 12.2.3. Limitations/Challenges

Despite its high accuracy, CCB-Detection requires specialized equipment for RNA handling, including thermocyclers and fluorometers, which may limit its adoption in resource-constrained laboratories. Food matrices containing RNases or inhibitory compounds (e.g., polyphenols in juices or proteins in milk) may interfere with RNA stability and enzymatic activity, necessitating rigorous sample preprocessing. The reliance on precise guide RNA design also demands prior knowledge of target sequences, potentially restricting its utility for novel or divergent strains [99].

### 12.3. DNA Microarray

#### 12.3.1. Objective and Methodology

This method detects and profiles virulence genes in *S. aureus* strains isolated from various foods. The methodology involves extracting genomic DNA from *S. aureus* isolates and fragments and labeling it with fluorescent or colorimetric markers. The labeled DNA is then hybridized to the microarray chip containing toxin-specific probes. Post-hybridization, signal detection is performed to identify the presence of target genes, with results visualized through imaging systems [5,93,100].

#### 12.3.2. Performance

The DNA Microarray successfully identified PVL, TSST-1, and various enterotoxin genes in most of the analyzed S. aureus isolates recovered from various foods (e.g., raw milk, retail meat, sausages, creamy cake, and rabbit carcasses), demonstrating its utility in toxin gene profiling [5,101,102]. However, sensitivity and specificity metrics were not reported, limiting direct comparisons with quantitative methods like PCR. The method’s ability to screen multiple genes concurrently offers an advantage over single-target assays, although its diagnostic accuracy remains undefined [103].

#### 12.3.3. Limitations/Challenges

Key challenges include cross-hybridization risks, where non-target DNA sequences may bind to probes, leading to false positive signals. The requirement for high-quality genomic DNA and optimized hybridization conditions further complicates its application in resource-limited settings. Additionally, the absence of sensitivity and specificity data raises concerns about its reliability in complex food matrices, which may contain inhibitors affecting hybridization efficiency. The subjective interpretation of signal intensity, particularly in colorimetric systems, may also introduce variability in results [93].

### 12.4. PCR-SSCP (Polymerase Chain Reaction–Single-Strand Conformation Polymorphism)

#### 12.4.1. Objective and Methodology

The primary objective of PCR-SSCP is to differentiate MRSA from MSSA strains by targeting genetic markers associated with resistance or virulence. The methodology begins with DNA extraction from bacterial isolates, followed by PCR amplification of conserved regions (e.g., 16S rRNA) or virulence-associated genes (e.g., *nuc*). Amplified products are denatured into ssDNA and subjected to electrophoresis under non-denaturing conditions. The resulting band patterns, which reflect sequence-specific conformations, are visualized and compared to reference strains. Distinct migration profiles allow for precise strain discrimination, facilitating epidemiological tracking during foodborne outbreaks [24,104].

#### 12.4.2. Performance

PCR-SSCP demonstrated 100% sensitivity and specificity in differentiating MRSA and MSSA among 159 staphylococcal isolates, outperforming MALDI-TOF-MS in strain resolution. Unlike MALDI-TOF-MS, which relies on protein mass spectrometry, PCR-SSCP detects subtle genetic polymorphisms, enhancing its utility for identifying clonal relationships and emerging resistance traits. However, its performance depends on selecting appropriate target genes and optimized electrophoretic conditions [24].

#### 12.4.3. Limitations/Challenges

A key limitation of PCR-SSCP is its reliance on technical expertise for interpreting complex band patterns, as minor variations in gel conditions or staining methods can affect reproducibility. The method is unsuitable for non-culturable samples due to its requirement for high-quality DNA, which necessitates prior bacterial cultivation. Additionally, the analysis is restricted to small DNA fragments (typically < 300 bp), as larger fragments may exhibit inconsistent migration due to secondary structures. Standardization challenges across laboratories further limit its widespread adoption, particularly in resource-limited settings. These constraints underscore the need for complementary methods in comprehensive outbreak investigations [24].

### 12.5. Plasmid Profiling

#### 12.5.1. Objective and Methodology

The primary aim of plasmid profiling is to investigate plasmid-associated antibiotic resistance in bacterial strains, particularly MRSA. In a study analyzing 13 MRSA strains isolated from meat samples, the methodology involved culturing bacterial isolates, followed by plasmid extraction using enzymatic or alkaline lysis protocols. The extracted plasmids were then subjected to agarose gel electrophoresis, where their sizes were inferred based on migration distances relative to DNA size markers. Strains were compared to identify variations in plasmid profiles, which reflect genetic diversity and potential horizontal gene transfer events [34].

#### 12.5.2. Performance

Plasmid profiling successfully identified plasmids in 12 of 13 MRSA isolates, highlighting its utility in detecting plasmid-borne genetic elements. However, sensitivity and specificity metrics were not reported, limiting quantitative comparisons with other molecular methods. The technique’s strength lies in its simplicity and cost-effectiveness for preliminary plasmid screening, although it lacks the resolution of advanced genomic approaches like whole-genome sequencing [34].

#### 12.5.3. Limitations/Challenges

A significant limitation of plasmid profiling is its inability to detect chromosomal resistance genes, restricting its scope to plasmid-mediated traits. The method requires pure bacterial cultures, making it unsuitable for direct application in complex food matrices without prior isolation. The subjective interpretation of gel electrophoresis results, particularly for plasmids of similar sizes, may lead to variability in data analysis. Additionally, plasmids can be spontaneously lost or acquired, leading to transient profile changes that may not reflect true genetic relatedness. These constraints emphasize the need for complementary techniques in comprehensive resistance profiling [34].

### 12.6. RAPD (Random Amplification of Polymorphic DNA)

#### 12.6.1. Objective and Methodology

The primary objective of RAPD in this context was to assess genetic diversity and clonality among *S. aureus* isolates derived from Koozeh cheese [44], poultry [105], and other food sources [106]. The methodology involved extracting genomic DNA from the isolates, followed by PCR amplification using random RAPD primers. Amplified DNA fragments were separated by agarose gel electrophoresis, and banding patterns were analyzed to infer genetic relationships. The absence of identical or highly similar profiles across isolates indicated no clonal relationships, suggesting significant genetic heterogeneity within the sampled population [107,108].

#### 12.6.2. Performance

RAPD successfully differentiated *S. aureus* isolates, revealing distinct genetic profiles and no evidence of clonality. However, sensitivity and specificity metrics were not reported, limiting the quantitative evaluation of its accuracy compared to standardized methods like MLST or PFGE [109]. The technique’s strength lies in its simplicity and cost-effectiveness for preliminary diversity assessments in settings lacking advanced genomic infrastructure [107,108].

#### 12.6.3. Limitations/Challenges

A major limitation of RAPD is its susceptibility to variability due to minor fluctuations in PCR conditions (e.g., annealing temperature and primer concentration), which can alter banding patterns and compromise reproducibility. The subjective interpretation of gel electrophoresis results, particularly for faint or overlapping bands, introduces potential bias [108]. Additionally, food matrices may harbor inhibitors (e.g., proteins and polysaccharides) that interfere with DNA amplification, reducing PCR efficiency [110]. The lack of sensitivity and specificity data further constrains its reliability for high-stakes applications such as outbreak tracing. These challenges necessitate cautious interpretation of results and complementary validation with more robust methods [107,108].

### 12.7. Rep-PCR (Repetitive Sequence-Based PCR)

#### 12.7.1. Objective and Methodology

The objective of Rep-PCR in this study was to group *S. aureus* isolates from meat samples of diverse geographic origins according to their genetic similarity. The methodology involved extracting genomic DNA from the isolates, followed by PCR amplification using primers designed to target conserved repetitive sequences. Amplified DNA fragments were separated via agarose gel electrophoresis, and the resulting banding patterns were digitized. Similarity coefficients were calculated based on band presence or absence, and dendrograms were constructed to visualize clustering patterns. Isolates with >95% similarity were grouped into clusters, facilitating strain classification [111,112].

The characterization and isolation of *S. aureus* using Rep-PCR have been reported in various food products, such as meat [34], raw poultry [113], and pastries [114]. This method has been proven to be effective in identifying the bacteria’s presence in different food matrices, highlighting its potential role in foodborne illnesses.

#### 12.7.2. Performance

Rep-PCR analysis revealed three major genetic clusters among *S. aureus* isolates from different countries, indicating high intragroup similarity despite geographic diversity. While the method effectively grouped strains based on genetic relatedness, sensitivity and specificity metrics were not reported, precluding direct comparisons with quantitative genotyping methods. Its utility lies in rapid and cost-effective strain differentiation, particularly in settings lacking advanced genomic infrastructure [34].

#### 12.7.3. Limitations/Challenges

A key limitation of Rep-PCR is its dependence on primer specificity and consistent PCR conditions, as minor variations in annealing temperature or primer concentration can alter banding patterns and reduce reproducibility. The requirement for bioinformatics tools to construct and interpret dendrograms may pose challenges in laboratories with limited computational resources. Additionally, the method does not provide insights into specific genetic determinants of virulence or antibiotic resistance, limiting its application in mechanistic studies. The subjective interpretation of banding patterns and dendrogram thresholds further complicates standardization across studies, necessitating complementary methods for comprehensive outbreak analysis [34,115].

### 12.8. MLVA (Multiple-Locus Variable-Number Tandem Repeat Analysis)

#### 12.8.1. Objective and Methodology

The primary objective of MLVA in this context was to perform high-resolution genetic typing of *S. aureus* isolates from food samples [89,116]. The methodology involved selecting multiple VNTR loci that exhibit repeat variability, followed by multiplex PCR amplification of these regions. Amplified fragments were separated and sized using capillary electrophoresis on an ABI 3500 genetic analyzer. The resulting fragment length data were converted into repeat numbers at each locus, and numerical profiles were compared to assess genetic relatedness. This approach allowed researchers to differentiate higher-resolution strains than traditional MLST [117].

#### 12.8.2. Performance

MLVA demonstrated superior discriminatory power compared to MLST, resolving genetic diversity among *S. aureus* isolates with greater precision. This enhanced resolution is attributed to the inherent variability of VNTR loci, which accumulate mutations more rapidly than housekeeping genes targeted by MLST. However, sensitivity and specificity metrics were not reported, limiting the quantitative evaluation of its diagnostic accuracy [118,119].

#### 12.8.3. Limitations/Challenges

A major limitation of MLVA is its reliance on prior knowledge of VNTR loci, which may not be well-characterized for all bacterial lineages or emerging strains. The method requires specialized equipment, such as capillary electrophoresis systems, and expertise in fragment analysis, restricting its use in resource-limited laboratories [120]. The standardization of protocols and interpretation criteria across studies remains challenging, as differences in locus selection or sizing methods can affect reproducibility. Additionally, MLVA profiles do not provide direct information on functional traits, such as virulence or antibiotic resistance, necessitating complementary methods for comprehensive strain characterization. These constraints underscore its role as a supplemental tool in outbreak investigations rather than a standalone diagnostic approach [89,121].

## 13. Biosensor-Based Methods

### 13.1. Objective and Methodology

Biosensor-based methods are designed to achieve rapid, sensitive, and specific detection of *S. aureus*, its enterotoxins, or other foodborne contaminants in complex matrices [122]. These methods prioritize minimizing sample pretreatment, reducing detection times, and surpassing the sensitivity of traditional techniques like ELISA or PCR [123].

The CNT-FET Biosensor targets the ultra-sensitive detection of SEC by employing carbon nanotubes functionalized with SEC-specific aptamers. The binding of SEC induces conformational changes in the aptamers, altering the nanotubes’ electrical conductivity, which is measured as a signal. This label-free approach enables detection limits as low as 1.25 fg/mL in food homogenates within 5 min, bypassing DNA extraction or amplification [124]. FCNB-based lateral flow assays (LFASs) utilize fluorescence-emitting carbon nanobeads conjugated to antibodies or aptamers to detect AFB1 and *S. aureus* simultaneously. Upon target binding, the fluorescent signal is amplified, achieving sensitivities of 95.5–99.3% and specificities of 97.3–98.5% in milk, ice cream, and meat samples. This methodology outperforms colorimetric LFAs by enhancing signal visibility and quantitation [125].

CD-MSN-based Lateral Flow Immunoassays integrate fluorescent carbon dots embedded in mesoporous silicon nanoparticles (CD-MSNs) to detect AFB1 and *S. aureus* through competitive or sandwich models. The probes are immobilized on test strips, where target binding triggers fluorescence proportional to analyte concentration, achieving detection limits of 0.05 ng/mL for AFB1 and 10 CFU/mL for *S. aureus*—significantly lower than gold nanoparticle-based methods [126]. Colorimetric and Fluorescence Dual-Mode Immunoassays combine p-phenylenediamine (PPD) for visual colorimetric detection and fluorescein for fluorescence readouts. PPD acts as a chromogen and fluorescence quencher, enabling rapid hemolysin (Hla) detection in dairy products with 97% sensitivity and 96% specificity [127].

Paper-based biosensors exploit *S. aureus*’s proteolytic activity by immobilizing a peptide substrate on magnetic nanobeads linked to a gold sensor. Protease cleavage releases nanobeads, causing a color change that is detectable visually or via ImageJ software. This method detects *S. aureus* in ground beef, milk, and environmental samples with 85% sensitivity and 100% specificity, eliminating the need for DNA extraction or PCR equipment [128]. Multicolor Time-Resolved Fluorescence Aptasensors employ lanthanide-doped nanoparticles (LnNPs) and graphene oxide (GO) for multiplex enterotoxin detection (SEA, SEB, and SEC1). Aptamer-conjugated LnNPs bind toxins, and fluorescence resonance energy transfer (FRET) between LnNPs and GO quenches the signal upon binding, achieving detection limits below 1 ng/mL in untreated milk [129].

Dual-Recognition SERS Biosensors enhance specificity by combining aptamer-functionalized magnetic nanoparticles (Fe3O4@Au MNPs) for bacterial capture and vancomycin-modified SERS tags for cell wall binding. After magnetic separation, SERS amplifies signals, enabling the detection of *S. aureus* at 3 cells/mL in 50 min—superior to culture-based methods [130]. Aptamer-based FLFAs further leverage FCNBs conjugated to aptamers for contaminant detection, achieving higher sensitivity than ELISA and HPLC without complex sample preparation [125]. Collectively, these methodologies integrate biorecognition elements, nanomaterials, and optical/electrochemical transduction to address the challenges of speed, sensitivity, and specificity in food safety monitoring.

### 13.2. Performance

Biosensor-based methods demonstrate exceptional sensitivity, specificity, and rapidity in detecting *S. aureus* and its toxins, outperforming traditional techniques in food safety monitoring. The CNT-FET Biosensor achieves an ultra-sensitive detection limit of 1.25 fg/mL for SEC in food homogenates, with high specificity and no cross-reactivity to analog toxins, delivering results within 5 min—significantly faster than ELISA or PCR [124]. Similarly, FCNB-based lateral flow assays (LFAs) exhibit sensitivities of 99.3% for AFB1 and 95.5% for *S. aureus*, alongside specificities of 98.5% and 97.3%, respectively, in complex matrices like milk, ice cream, and meat, leveraging fluorescence amplification to surpass colorimetric LFAs [125].

Advanced platforms such as CD-MSN-based Lateral Flow Immunoassays detect AFB1 at 0.05 ng/mL and *S. aureus* at 10 CFU/mL, achieving detection limits far below conventional gold nanoparticle-based methods [126]. The Multicolor Time-Resolved Fluorescence Aptasensor further enhances multiplex detection, identifying enterotoxins SEA, SEB, and SEC1 at 0.062 ng/mL, 0.069 ng/mL, and 0.040 ng/mL, respectively, in untreated milk, with minimal cross-reactivity (<15%) [129]. For bacterial detection, the Dual-Recognition SERS Biosensor achieves an unprecedented limit of 3 cells/mL for *S. aureus* using surface-enhanced Raman scattering, coupled with 106.4% recovery, outperforming culture-based methods in both sensitivity and speed [130].

These methods also excel in simplicity and speed. Paper-based biosensors detect *S. aureus* in <2 h with 85% sensitivity and 100% specificity in ground beef and milk, eliminating the need for DNA extraction or specialized equipment [128]. Colorimetric and Fluorescence Dual-Mode Immunoassays provide simultaneous visual and instrumental readouts in <1 h, achieving 97% sensitivity and 96% specificity for hemolysin detection in dairy products [127].

Despite these advantages, challenges persist. Food matrix components, such as fats and proteins, can interfere with fluorescence signals or block biorecognition sites, necessitating sample pretreatment [130]. Additionally, reliance on specialized equipment (e.g., fluorescence readers and SERS detectors) may limit field applicability, while the high cost of nanomaterials like lanthanide-doped nanoparticles or FCNBs poses scalability barriers [125,128]. Nevertheless, biosensors represent a transformative advancement in foodborne pathogen detection, balancing rapidity, precision, and practicality for outbreak investigations.

### 13.3. Limitations/Challenges

Despite their advantages, biosensor-based methods face several challenges that hinder their universal application in foodborne pathogen detection. Food matrix interference remains a critical limitation, as components like fats, proteins, or particulate matter in complex samples (e.g., dairy products and meat homogenates) can quench fluorescence signals, block biorecognition sites, or generate false positives. For instance, the Dual-Recognition SERS Biosensor requires meticulous sample homogenization to mitigate interference from food debris, complicating rapid field deployment [130]. Similarly, fluorescence-based assays, such as FCNB-based LFAs, may suffer reduced sensitivity in fatty matrices like ice cream or processed meats due to light scattering or autofluorescence [125].

Specialized equipment dependency further limits practicality. Methods like Multicolor Time-Resolved Fluorescence Aptasensors or SERS-based platforms necessitate advanced instrumentation (e.g., fluorescence readers and Raman spectrometers) for signal detection, restricting their use in resource-limited settings or field applications [129,130]. Paper-based biosensors, while portable, still require imaging tools like ImageJ software for quantitative analysis, reducing their standalone utility [128].

The cost and scalability of nanomaterials pose additional barriers. High-purity carbon nanotubes (CNTs), lanthanide-doped nanoparticles, or fluorescent carbon nanobeads (FCNBs) are expensive to synthesize, limiting large-scale production and commercialization [125]. Furthermore, the stability of biorecognition elements (e.g., aptamers and antibodies) under varying storage conditions (e.g., temperature and humidity) remains unaddressed, risking degradation and reduced shelf life [124].

Sensitivity trade-offs are evident in certain applications. For example, the paper-based biosensor achieves 85% sensitivity for *S. aureus* detection in food samples, which is lower than that of PCR-based methods, potentially missing low-level contaminations in outbreak scenarios [128]. Similarly, Dual-Mode Immunoassays prioritize rapidity but lack multiplexing capabilities, restricting the simultaneous detection of multiple pathogens or toxins [127].

Finally, validation gaps persist. Many biosensors are validated in controlled laboratory settings using spiked samples rather than real-world contaminated foods, raising concerns about performance under natural contamination levels or microbial competition [126]. The standardization of protocols across diverse food matrices remains unresolved, complicating regulatory acceptance and harmonization with existing food safety frameworks [129].

## 14. AI-Based Methods

### 14.1. Objective and Methodology

The primary objectives of AI-driven methodologies for detecting *S. aureus* in food are to achieve rapid, accurate, and automated identification of the pathogen and its antibiotic-resistant variants while overcoming limitations of traditional techniques such as prolonged processing times, labor-intensive workflows, and susceptibility to food matrix interference. These methods leverage diverse technological platforms integrated with machine learning (ML) and deep learning (DL) models to enhance specificity, sensitivity, and scalability in complex food samples [131,132].

Fluorescence-based approaches, such as color-encoded multiplex hydrogel digital LAMP, target antibiotic resistance genes (e.g., *mecA* for MRSA) using primers labeled with distinct fluorophores (red, green, and blue). DNA amplicons produce fluorescent spots during amplification, which AI algorithms automatically quantify to estimate bacterial loads, enabling multiplex detection in a single test [131]. Similarly, single-stranded DNA (ssDNA) sensor arrays employ fluorescence-labeled probes that recover emission upon binding to bacterial targets, generating unique spectral fingerprints. These patterns are classified by ML models like multilayer perceptrons (MLPs) and Kolmogorov–Arnold Networks (KANs), achieving 93.8–98.4% accuracy after 30–120 min of incubation, significantly outperforming traditional ELISA in speed and multiplex capability [133].

Genomic methods combine WGS with ML to predict AMR. By analyzing k-mers (short DNA sequences) from *S. aureus* isolates, models such as radial basis function support vector machines (RBF-SVMs) classify resistance profiles with >90% sensitivity and specificity for antibiotics like oxacillin, providing insights into the genetic determinants of resistance that are often missed by conventional diagnostics [134]. Microfluidic systems integrate time-lapse imaging of bacterial growth in microwells with DL architectures like the Time-Lapse Images Driven Efficient Net-Transformer Network (TLENTNet), which extracts spatiotemporal features to enumerate viable *S. aureus* at 63 CFU/mL within 7 h, demonstrating 97.72% accuracy [135].

Spectroscopic techniques, such as SERS, amplify bacterial spectral signals using nanostructured substrates (e.g., Au/Ag nanoflowers modified with iodide ions). ML models like CNNs decode these spectra to distinguish MRSA from MSSA based on subtle spectral markers, enabling label-free and real-time detection without culturing [132,136]. Hybrid platforms, including SERS/PCR biosensors, merge nucleic acid amplification with ML spectral analysis (e.g., BOSS-PLS algorithms), achieving high sensitivity (Rp = 0.967) for *S. aureus* gene targets in milk [132].

Morphological classification via CNNs automates *S. aureus* recognition in microscopic images by training on datasets of cocci clusters, achieving 90–100% accuracy and reducing human error inherent in manual inspection [137]. Indirect detection methods, such as ML analysis of gas emissions (e.g., CO_2_) from meat samples, correlate metabolic byproducts with bacterial growth. Advanced Random Forest models excel in this approach, offering non-invasive contamination monitoring [138]. Predictive modeling using ANN simulates *S. aureus* growth and enterotoxin production in dairy products like Kazak cheese, incorporating parameters such as water activity and fermentation temperature to predict risks with R-values up to 0.976, surpassing the logistic regression model [139].

Collectively, these methodologies emphasize automation and data-driven decision-making, replacing subjective interpretation with AI-optimized thresholds. They address food matrix challenges through tailored preprocessing steps and enhance scalability in food safety workflows. They offer transformative advantages over traditional methods like plate counting or PCR, which lack multiplex capabilities and require extensive manual intervention [133,136].

### 14.2. Performance

AI-driven methods exhibit exceptional performance in detecting *S. aureus*, characterized by high accuracy, sensitivity, and rapid turnaround times, outperforming traditional techniques. Fluorescence-based approaches, such as color-encoded digital LAMP, achieve multiplex detection of antibiotic-resistant strains within hours, which is significantly improved compared to culture-based methods requiring days. The AI quantification of fluorescent spots demonstrates precision in correlating spot counts with bacterial loads, validated in real food samples like fruits and vegetables [131].

ssDNA sensor arrays paired with ML classifiers, such as MLP and KAN, achieve 93.8% accuracy after 30 min of incubation, escalating to 98.4% at 120 min, surpassing ELISA in both speed and multiplex capability [133].

Genomic ML models analyzing WGS data demonstrate robust predictive power for AMR. Radial basis function support vector machines (RBF-SVMs) achieve >90% sensitivity and specificity for antibiotics like oxacillin, accurately linking k-mer patterns to resistance phenotypes across 673 *S. aureus* isolates [134]. Microfluidic systems integrated with deep learning, such as TLENTNet, enumerate *S. aureus* at 63 CFU/mL within 7 h with 97.72% accuracy, out performing plate counting in sensitivity and speed [135].

Spectroscopic techniques, including SERS-ML platforms, distinguish MRSA from MSSA via spectral fingerprints with high precision, leveraging CNNs to decode subtle spectral markers undetectable by conventional methods [136]. Hybrid SERS/PCR biosensors achieve near-perfect correlation (Rp = 0.967) in detecting *S. aureus* gene targets, which are validated in milk samples [132].

Morphological classification via CNNs trained on microscopic images attains 90–100% accuracy in identifying *S. aureus* cocci clusters, reducing human error inherent in manual microscopy [137]. Indirect detection methods, such as Advanced Random Forest models analyzing gas emissions from meat samples, show superior performance correlating metabolic activity with bacterial contamination, out performing SVM and ANN in precision [138]. Predictive ANN models simulate *S. aureus* growth and enterotoxin production in dairy products with R-values of 0.918–0.976, surpassing logistic regression in forecasting risks under variable environmental conditions [139].

Comparative studies highlight AI’s superiority: MLP neural networks in ssDNA arrays achieve 98.4% accuracy post-incubation, while traditional plate counting and ELISA lack comparable specificity and require days for results [133,136]. AI methods also excel in multiplex detection, simultaneously identifying multiple pathogens (e.g., *S. aureus* and *Salmonella*) in complex matrices like milk, where conventional techniques struggle with cross-reactivity [133]. However, performance variability exists; for instance, fluorescence-based systems may require preprocessing to mitigate food matrix interference, and CNNs depend on high-quality annotated image datasets [133,137]. Despite these challenges, AI-driven approaches consistently demonstrate faster detection limits (e.g., 7 h for microfluidics vs. 24–48 h for culturing) and higher throughput, making them indispensable for rapid outbreak response [131,135].

### 14.3. Limitations/Challenges

Despite their advantages, AI-driven methods face several challenges that hinder widespread adoption. Complex food matrices, such as dairy and meat, introduce interference by quenching fluorescence signals (e.g., fats in dairy obscuring fluorophore emissions) or masking spectral fingerprints in SERS, necessitating preprocessing steps like filtration or chemical treatments. However, these steps risk removing target bacteria or altering sample integrity, particularly in heterogeneous food samples [133,136]. Technical dependencies on specialized equipment, such as nanostructured SERS substrates (e.g., Au/Agnanoflowers) or microfluidic chips, pose barriers due to high fabrication costs, batch variability, and the need for advanced laboratory infrastructure, limiting accessibility for resource-constrained settings [132,135].

AI models require large annotated datasets for training, which are labor-intensive to compile. Genomic ML approaches depend on expertly labeled WGS data. CNNs for microscopic imaging demand high-quality images with consistent annotations, which may vary due to differences in staining protocols or microscope calibration [134,137]. Furthermore, datasets often lack diversity across food matrices and bacterial strains, raising concerns about model generalizability. For instance, ML models trained on *S. aureus* isolates from one food type may underperform in others, necessitating retraining or transfer learning [134].

Subjectivity in manual annotation introduces biases, as inconsistencies in labeling microscopic images or spectral data by different experts can skew model performance. Standardized protocols for data collection and annotation are critical but not always feasible in practice [137]. Additionally, some sensor-based methods, such as ssDNA arrays, require extended incubation periods (30–120 min) to achieve optimal accuracy, which may delay time-sensitive decisions in food production environments [133].

While AI models excel in multiplex detection, cross-reactivity remains risky, where non-target bacteria or food components generate false positive signals. Although advanced feature extraction in ML algorithms mitigates this, validation in real-world samples is essential [133]. Environmental factors, such as temperature fluctuations during gas emission analysis or microfluidic operation, may also affect reproducibility, demanding stringent control measures unsuitable for field applications [138].

Finally, high computational costs for training DL models and the “black-box” nature of AI systems complicate troubleshooting and regulatory approval, as transparency in decision-making is often required for compliance with food safety standards [136,139].

## 15. Conclusions

The detection of *S. aureus* and its enterotoxins in foodborne outbreaks has evolved significantly, transitioning from culture-dependent assays to rapid molecular and biosensor-based technologies. While immunological and PCR methods remain cornerstones for their specificity and scalability, emerging tools like CRISPR-based assays and sequencing offer transformative potential for precision and outbreak tracking. However, persistent challenges—such as matrix interference, inability to confirm toxin activity genetically, and resource disparities—highlight the need for context-appropriate method selection. The integration of techniques, such as combining PCR with ELISA or leveraging biosensors for initial screening, followed by sequencing for confirmation, could mitigate individual limitations. Future advancements must prioritize standardized reporting, cost-effective automation, and innovations to address viability assessment and matrix complexity. Collaborative efforts among researchers, industry, and regulators are essential to harmonize protocols and accelerate the adoption of next-generation diagnostics, ultimately strengthening global food safety systems against *S. aureus*-related threats.

## Data Availability

No new data were created or analyzed in this study.
